# A High-Performance and Low-Cost Soy Flour Adhesive with a Hydroxymethyl Melamine Prepolymer

**DOI:** 10.3390/polym10080909

**Published:** 2018-08-13

**Authors:** Meng Zhang, Yi Zhang, Mingsong Chen, Qiang Gao, Jianzhang Li

**Affiliations:** 1 Key Laboratory of Wood Material Science and Utilization, Beijing Forestry University, Beijing 100083, China; zhangmeng@bjfu.edu.cn (M.Z.); luckyyizhang@163.com (Y.Z.); chen_boss@bjfu.edu.cn (M.C.); 2 Beijing Key Laboratory of Wood Science and Engineering, Ministry of Education, College of Materials Science and Technology, Beijing Forestry University, Beijing 100083, China

**Keywords:** soy flour adhesive, hydroxymethyl melamine prepolymer, polysaccharose, interpenetrating network structure, water resistance

## Abstract

To improve the performance of a soy flour (SF)-based adhesive, a low-cost hydroxymethyl melamine prepolymer (HMP) was synthesized and then used to modify the SF-based adhesive. The HMP was characterized, and the performance of the adhesive was evaluated, including its residual rate, functions, thermal stability, and fracture section. Plywood was fabricated to measure wet shear strength. The results indicated that the HMP preferentially reacted with polysaccharose in SF and formed a cross-linking network to improve the water resistance of the adhesive. This polysaccharose-based network also combined with the HMP self-polycondensation network and soy protein to form an interpenetrating network, which further improved the water resistance of the adhesive. With the addition of 9% HMP, the wet shear strength (63 °C) of the plywood was 1.21 MPa, which was 9.3 times that of the SF adhesive. With the HMP additive increased to 15%, the shear strength (100 °C) of the plywood was 0.79 MPa, which met the plywood requirement for exterior use (≥0.7 MPa) in accordance with Chinese National Standard (GB/T 9846.3-2004). With the addition of 9% and 15% HMP, the residual rates of the adhesive improved by 5.1% and 8.5%, respectively. The dense interpenetrating network structure improved the thermal stability of the resultant adhesive and created a compact fracture to prevent moisture intrusion, which further increased the water resistance of the adhesive.

## 1. Introduction

The soy-based adhesive is a green and ecofriendly adhesive that provides several advantages, including rich and cheap sources of raw materials, ease of handling, and renewability [1,2,3]. Studies have been conducted on the soy-based adhesive as a replacement for the formaldehyde-based adhesive, and its use in the wood-based panel fabrication industry [4,5]. However, the soy-based adhesive also has several disadvantages, including high viscosity, low bonding strength, and poor water resistance, which limit its industrial applications [6,7].

A number of studies have been conducted to improve the water resistance of the soy protein-based adhesive by modification [8], such as molecular modification and denaturing agent cross-linker modification [9]. Soy protein molecular modification is mainly conducted by grafting highly active groups onto soy protein molecules to form a cross-linked network in the resultant adhesive after curing to improve water resistance [10,11,12]. This technique improves the wet shear strength of the resultant plywood; however, it is costly and complex, rendering it impractical for plywood fabrication. Denaturants unfold the protein molecule and create a more crystalline domain to prevent water intrusion, which enhances the water resistance of the resultant adhesive [13]. This increase in water resistance is insufficient though, and the plywood bonded with these modified adhesives containing denaturants fail to meet the plywood requirements for interior use [14]. (These requirements (Type II plywood) are determined in accordance with Chinese National Standards (GB/T9846.3-2004)). The cross-linker, such as epoxide and polyamidoamine-epichlorohydin (PAE) resin, can effectively improve the water resistance of soy-based adhesives, and the performance of the resultant plywood meets the interior use plywood requirements [15,16,17,18]. However, the plywood with the cross-linker-modified adhesive has dry shear strength lower than that of commercial formaldehyde-based adhesives.

Our previous research [19,20,21] identified the following reasons for the low dry bond strength of the soy protein adhesive: (1) The soy protein was characterized by a high molecular weight and could hardly penetrate the wood to form an interlock; (2) the function groups on soy protein exhibited low reactivity, which could hardly form a molecular force with the wood surface; and (3) as a main raw material, soy flour (SF) consisted of 38 wt % polysaccharose, which caused weak interface layers. Therefore, a small molecule modifier should be chosen and introduced into the soy-based adhesive system to improve both water resistance and dry bond strength.

In the present study, a hydroxymethyl melamine prepolymer (HMP) was synthesized using melamine (M) and formaldehyde (F) to obtain low-cost and water-soluble small-molecule prepolymers. The HMP as a cross-linker was mixed with SF to develop an SF–based adhesive. The functional groups, residual rate, thermal stability, and microscopic morphology of the adhesive were measured. A three-ply plywood was fabricated using the resultant adhesive, and its wet shear strength was tested. The bond strength and residual rate of the resultant adhesives were compared with those of the SF/PAE adhesive and the soy protein isolate (SPI)/HMP adhesive.

## 2. Materials and Methods

### 2.1. Materials

SF (46% soy protein, 38% polysaccharose, and other components) was purchased from Xiangchi Grrain and Oil Co. in Zibo, Shandong Province of China and milled into 200-mesh flour by using a grinder. SPI with 95% protein content, which was supplied by Yuwang Ecological Food Industry Co., Ltd. (Dezhou, Shandong, China), was also milled into 200-mesh flour. PAE resin was provided by Wuxi Tianxin Chemical Co., Ltd. (Wuxi, Jiangsu, China). HMPs were synthesized in the laboratory. Sodium hydroxide (NaOH), M, and F, which were purchased from Tianjin Chemical Reagent Co. (Tianjin, China), were of analytical grade. Other chemical reagents used in this study were analytical reagent-grade and provided by Beijing Chemical Reagents Co. Ltd., (Beijing, China). Poplar (*Populus tomentosa* Carr.) veneers (40 cm × 40 cm × 1.5 cm, 8% of moisture content) were supplied by Wen’an County Fujia Wood Industry Co., Ltd., (Wenan, Hebei, China).

### 2.2. Preparation of HMP

The overall F-to-M molar ratio that was used to prepare HMP was 2:1. M was added in two steps. First, F 37% (200 g) was added into a three-necked flask equipped with a mechanical stirrer and then adjusted to pH 9.0–9.5 with NaOH 20%. Second, M (100 g) was added, and the temperature was increased to 85 °C in 30 min while the reaction continued for 30–40 min at pH ≥ 9.0. Third, more M (55 g) was added to continue the reaction for 20–30 min. The mixture was finally cooled to 40 °C, and pH was kept within the 9.5–10.0 range. The resultant prepolymers were spray-dried to remove the free F, and a white powder was obtained. The HMP and water were mixed at a ratio of 1:1 to obtain a stable solution and then used to modify the soy-based adhesive.

### 2.3. Preparation of SF Adhesives

To prepare various adhesives, water was added with different contents of HMP and then mechanically stirred for 2 min at 25 °C. SF was then added and mechanically stirred for 3 min to obtain the homogeneous adhesive. Detailed formulations of the adhesive are shown in Table 1.

The HMP (sample H) adhesive was prepared as the control: 20 g wheat flour (WF) (as the filler) was added to 80 g HMP solution (with 50% water) and then mechanically stirred for 5 min at 25 °C. In addition, the SF/PAE adhesive (sample P) was prepared using the following procedure: SF (25 g) was added into the mixture of deionized water (25 g) and PAE solution (concentration 12%, 50 g), and then mechanically stirred for 5 min at 25 °C.

To further clarify the reaction between HMP and SF, the same method was used to prepare the control adhesive, with SPI as the raw material. Protein comprised 13.3% of the SPI adhesives to ensure that all adhesives had the same protein content. Detailed formulations of the adhesive are shown in Table 2.

### 2.4. Preparation of Three-Plywood Samples

A three-ply poplar plywood was fabricated in this study. The optional conditions for the manufacture of three-plywood samples are as follows: Adhesive loading, 180 g/m^2^ (evenly spread on a single plywood surface); hot-press temperature, 122 °C; hot press pressure, 1.0 MPa; and hot-press time, 70 s/mm. The water content of the resulting plywood after hot-pressing ranged from 8–10%. The plywood samples were stored under ambient conditions for at least 24 h before testing.

### 2.5. Characterization of Adhesive Samples

#### 2.5.1. Shear Strength Measurement of Plywood

**Figure 1:**
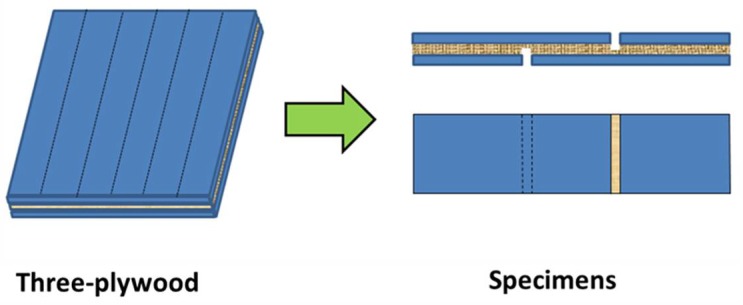
Preparation of the specimens for shear strength testing.

The properties of the bonded plywood were determined using the shear strength test. Figure 1 presents the preparation of the specimens for the shear strength test. First, the bonded plywood was cut into specimens measuring 100 mm × 25 mm (gluing area of 25 mm × 25 mm) [19]. The cut specimens were then placed in a dry room, immersed in water at 63 °C, and boiled at 100 °C and remained under these three conditions for 3 h. The shear strength was finally determined using a common tensile machine operating at a speed of 10.0 mm·min^−1^ and then dried at room temperature for 10 min before testing. Each sample with six replicates was tested. In addition, when shear dry strength was measured, the samples were first wetted with a brush to reduce the destruction of the wood. The force required to break the glued specimen was recorded, and the average of each set of data was determined. The shear strength was calculated using the following equation:

(1) $$\tau_{max}=\frac{F_{max}}{A}$$

where $\tau_{max}$ is the shear strength, $F_{max}$ is the maximum force to break the glued specimen, and A is the gluing area (25 mm × 25 mm).

#### 2.5.2. Residual Rate Test

Residual rate testing was conducted to determine the hydrolytic stability of the adhesives. The samples were placed in an oven at 120 °C ± 2 °C until a constant weight (M) was obtained. The dried adhesive samples were crushed using a pestle, and the resulting products were 100–200 mesh. The cured adhesives weighing 1 g per sample and covered with filter paper were soaked in water (a glass beaker with 500 mL of water and heated in the water bath) at 50 °C for 4 h and then oven-dried again at 105 °C ± 2 °C for about 5 h until a constant weight was obtained (*m*). The residual rate of each sample was calculated from the three parallel values. The residual rate value (*m*/M) is reported as a percentage in this study, as in the following equation:

(2) $$\mathrm{Residual}\;\mathrm{rate}\;\left(\%\right)=\frac{m}{M}\;\times 100$$

where m and M are the weight of soaked in water at 50 °C for 4 h and weight before soaking, respectively.

#### 2.5.3. Fourier Transform Infrared (FTIR) Spectroscopy

The adhesive samples were cured in an oven at 120 °C ± 2 °C until a constant weight (M) was obtained. The dried adhesives were then individually ground into 200-mesh powder. Each powder sample was mixed with KBr crystals at a mass ratio of 1/70 and then pressed in a pellet for analysis. The FTIR spectra of the various adhesive samples were recorded on a Nicolet 6700 spectrometer (Nicolet Instrument Corporation, Madison, WI, USA) within the range of 400–4000 cm^−1^ with a 4 cm^−1^ resolution using 32 scans.

#### 2.5.4. Thermogravimetry (TGA) Test

The adhesive samples were cured in an oven at 120 °C ± 2 °C until a constant weight (M) was obtained and ground into 200 mesh. TG measurements were recorded on a TGA instrument (TA Q50, Waters Company, New Castle, DE, USA). About 5 mg powdered sample was weighed in a platinum cup and scanned from 10 °C to 610 °C at a heating rate of 10 °C/min in a nitrogen environment while recording the weight changes in the sample.

#### 2.5.5. Scanning Electron Microscopy (SEM) Analysis

The different adhesive samples (placed on an aluminum foil) were cured in an oven at 120 °C ± 2 °C until a constant weight (M) was obtained. The cured adhesives were broken, and the fracture surface of the samples was sputter-coated with gold using an E-1010 Hitachi ion sputter coater (Hitachi Science System, Ibaraki, Japan). We then observed the fractured surface of the adhesives by SEM (Hitachi Science System, Ibaraki, Japan).

## 3. Results and Discussion

### 3.1. FTIR Spectra of M and HMP

**Figure 2:**
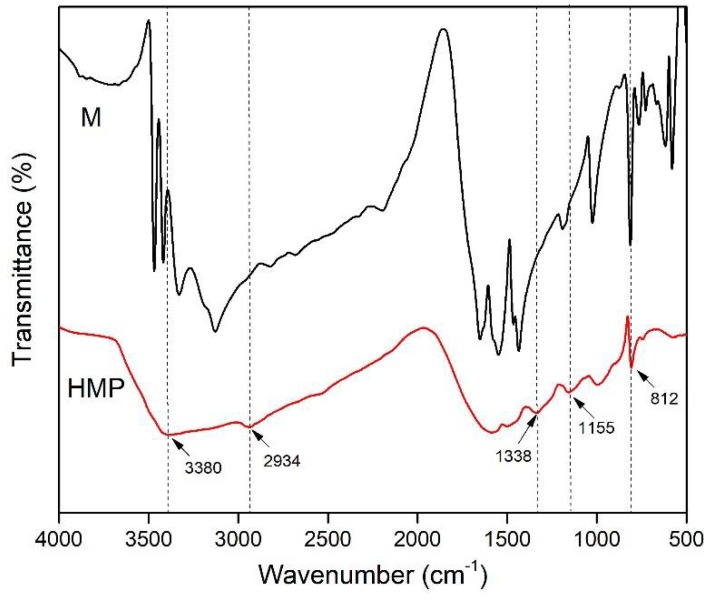
FTIR spectra of M and HMP.

**Figure 3:**
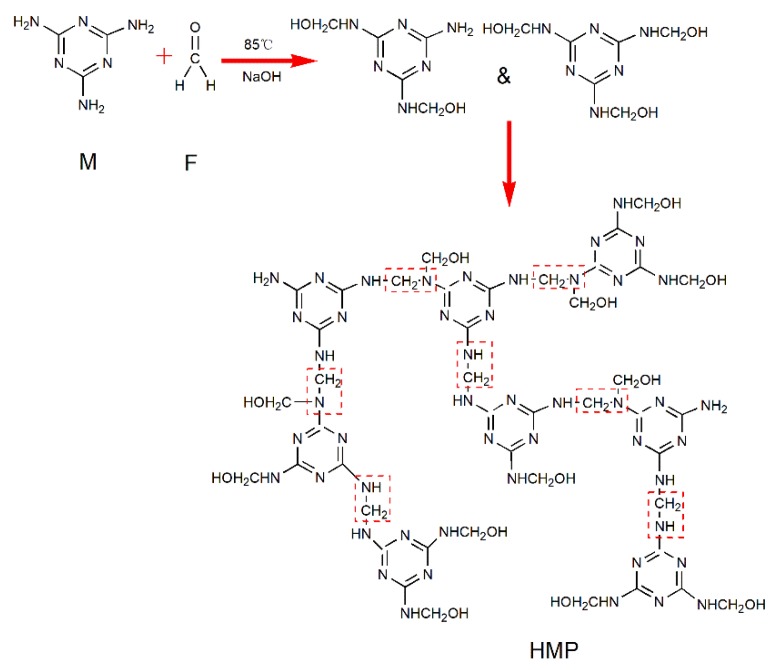
Chemical reaction synthesis of HMP.

The FTIR spectra of M and HMP are presented in Figure 2. Compared with the FTIR spectra of M, that of HMP showed a new absorption at 2934 cm^−1^, which was attributable to the –CH_2_ stretching vibrations [22,23]. In addition, the new peak observed at 1338 cm^−1^ correspond to the C–N stretching of CH_2_–N [24]. Both of them indicated that the –NH_2_ of the M reacted with formaldehyde to generate prepolymers. The appearance of the 1155 cm^−1^ band in the HMP spectra was assigned to the C–N stretching of the secondary amine [25], further suggesting the existence of the abundant –NH–CH_2_–NH– group. The M and HMP spectra exhibited the peak at 812 cm^−1^, which was assigned to the typical stretching of the triazine ring [26]. This finding indicated that M was introduced into the HMP system. The aforementioned results suggested that the HMP was successfully synthesized. Figure 3 shows the chemical reaction synthesis of HMP.

### 3.2. Tensile/Shear Strength Measurement

**Figure 4:**
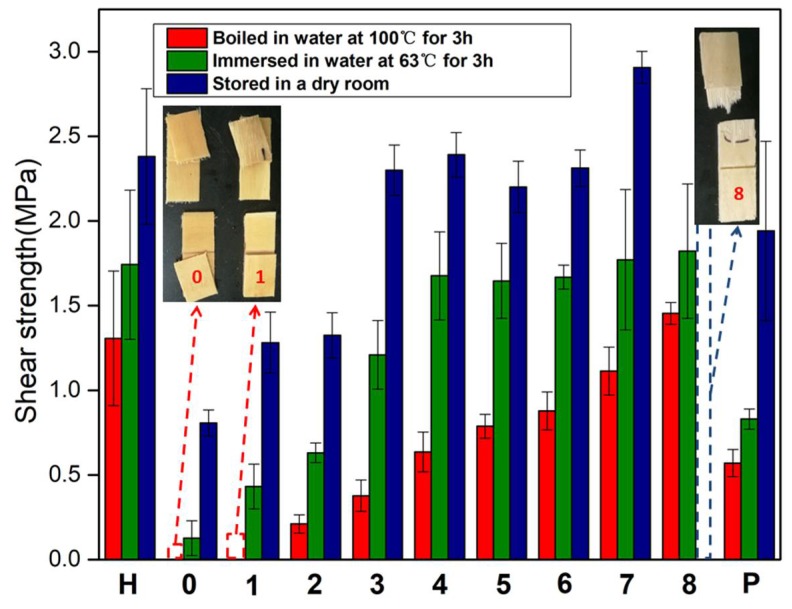
Shear strengths of the plywood bonded with the different adhesives: H (HMP adhesive), 0 (SF adhesive), 1 (SF/3% HMP adhesive), 2 (SF/6% HMP adhesive), 3 (SF/9% HMP adhesive), 4 (SF/12% HMP adhesive), 5 (SF/15% HMP adhesive), 6 (SF/18% HMP adhesive), 7 (SF/21% HMP adhesive), 8 (SF/24% HMP adhesive), and P (SF/PAE adhesive).

The effects of HMP content on the shear strength of the plywood bonded with the different adhesives under all testing conditions are shown in Figure 4. As shown in Figure 4H, the wet shear strength of the plywood bonded with the HMP adhesive immersed in water at 63 °C and boiled in water at 100 °C were 1.31 and 1.74 MPa, respectively. The excellent water resistance was attributable to the denser structure of the HMP adhesive. The shear strength of the plywood under all conditions increased with an increase in HMP additive into the adhesive formulation. The shear strengths of the plywood bonded with the SF adhesive in a dry room was 0.81 MPa and that immersed in water at 63 °C was 0.13 MPa. All specimens (plywood bonded with SF adhesive and SF/3% HMP adhesive) boiled in water at 100 °C were delaminated, and their shear strength could not be measured. When the HMP additive reached 9%, the dry strength of the plywood increased by 184%, resulting in 2.30 MPa. The shear strength of the specimens immersed in water at 63 °C was improved by 830% relative to that of the SF adhesive, resulting in 1.21 Mpa. And the SF/9% HMP adhesive exhibited a higher wet shear strength (63 °C) than other reports [18,27,28,29]. In addition, with the addition of 6% HMP, the plywood exhibited a shear strength of 0.21 MPa when boiled in water at 100 °C. With the HMP additive further increased to 24%, the specimens immersed in water at 63 °C and boiled in water at 100 °C exhibited shear strengths increased by 189% and 590%, respectively, relative to that of SF/6% HMP, thus reaching 1.82 and 1.45 MPa. The shear strength of the plywood (bonded with SF/24% HMP adhesive) stored in a dry room could not be measured because wood failed in all specimens during testing. With an HMP additive at 9%, the shear strength of the plywood immersed in water at 63 °C was 1.21 MPa, which met the plywood requirement for interior use. With an HMP additive of 15%, the specimen cooked in water at 100 °C exhibited a shear strength of 0.79 MPa, which met the plywood requirement for exterior use. The water resistance of the plywood for exterior use (Type I plywood) was determined using a tensile/shear strength tester (WDW-200E, Jinan Times Test Gold Testing Machine Co., Ltd., Jinan, China) in accordance with the Chinese National Standards (GB/T9846.3-2004)). This significant increase could be attributed to the (1) reaction of the HMP with the function groups of SF, and the formation of the cross-link network, which improved the water resistance of the adhesive, and (2) condensation polymerization of the HMP itself during hot pressing. During this process, a chain of large molecules formed an interpenetrating network, which further improved the bond performance of the adhesive. As shown in Figure 4P, the plywood bonded with the SF/PAE adhesive immersed in water at 63 °C exhibited a shear strength of 0.83 MPa, whereas that placed in a dry room had a shear strength of 1.67 MPa. However, with 9% HMP additive, the SF/HMP adhesive exhibited a better performance than that of the SF/PAE adhesive.

**Figure 5:**
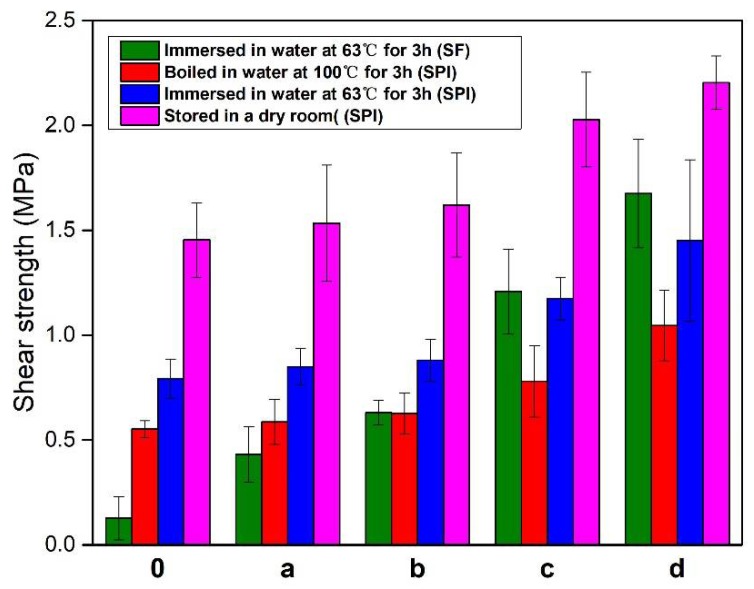
Shear strengths of SF and SPI adhesives: 0 (SF and SPI adhesives); a (SF/3% HMP and SPI/3% HMP adhesives); b (SF/6% HMP and SPI/6% HMP adhesives); c (SF/9% HMP and SPI/9% HMP adhesives); d (SF/12% HMP and SPI/12% HMP adhesives).

SF contains 38% polysaccharose, in addition to 46% soy protein, which significantly effects on the properties of the adhesive [20]. To evaluate the effect of polysaccharose on the water resistance of the SF/HMP adhesives, SF- and SPI-based adhesive with HMP at different concentrations was developed, and the resultant plywood was tested. Figure 5 shows the shear strength of the plywood bonded with the different adhesives. The wet shear strength (63 °C) of the plywood bonded with the SPI adhesive was 0.79 MPa, which was 5.1 times higher than that of the SF adhesive (0.13 MPa). Polysaccharose in SF was hydrophilic; thus, its existence reduced the water resistance of the adhesive. When the HMP additive was from 0% to 6%, the wet shear strength (63 °C) of the plywood bonded with the SF/HMP adhesive increased by 485% relative to that of the SF adhesive, reaching 0.63 MPa. However, with an HMP additive of 6%, no apparent change was observed in the wet shear strength (63 °C) of the plywood bonded with the SPI/HMP adhesives. A further increase in HMP additive to 9% led to increases in the wet shear strength (63 °C) of the SF/HMP and SPI/HMP adhesives relative to those of the SF and SPI adhesives. The increases were 830% and 48%, reaching 1.21 and 1.17 MPa, respectively. With 12%HMP, the wet shear strength (63 °C) of the plywood bonded with the SF/HMP adhesive was 1.68 MPa, which was 1.16 times that of the wet strength of the SPI/12%HMP adhesive (1.45 MPa). This result indicated that HMP reacted preferentially with polysaccharose in the SF rather than with the soy protein. For the SPI/HMP adhesive, when the HMP additive was 6% to 12%, the increase in the wet shear strength (63 °C) of the plywood was attributed to the self-polycondensation of the HMP and the formation of an interpenetrating network. In addition, as shown in Figure 5, the change in shear strength (under dry condition and when immersed in water at 63 °C and 100 °C) of the plywood bonded with the SF/SPI adhesives exhibited similar trends.

On the basis of the aforementioned analysis, the HMP reacted primarily with polysaccharose to form a dense network system to improve the water resistance of the SF-based adhesive. In addition, the HMP self-crosslinked to form a rigid HMP network when the HMP additive reached a certain level. This HMP network was uniformly distributed in the SF adhesive system and penetrated with the soy protein to form a penetrated network. This dense penetrated network blocked the entry of water and significantly improved the water resistance of the adhesive.

### 3.3. Residual Rate

**Figure 6:**
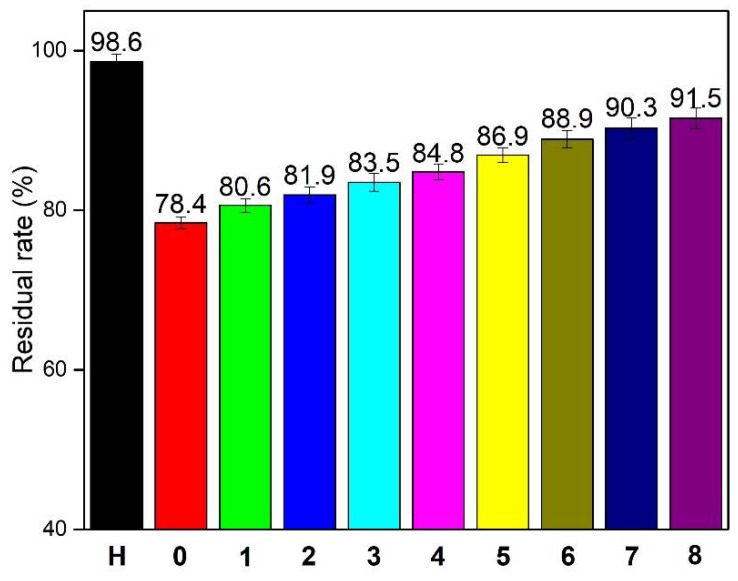
Residual rates of SF adhesives with different amounts of HMP additives: H (HMP adhesive), 0 (SF adhesive), 1 (SF/3% HMP adhesive), 2 (SF/6% HMP adhesive), 3 (SF/9% HMP adhesive), 4 (SF/12% HMP adhesive), 5 (SF/15% HMP adhesive), 6 (SF/18% HMP adhesive), 7 (SF/21% HMP adhesive), and 8 (SF/24% HMP adhesive).

For wood adhesives, the water resistance is an important property that can be measured using the residual rate [30]. Figure 6 shows the residual rates of the different SF adhesives. The residual rates of HMP and SF adhesive were 98.6% and 78.4%, respectively. HMP formed a dense structure by self-crosslinking, which exhibited the excellent water resistance. During hot pressing, the SF adhesive lost water and formed a hydrogen bond to produce bond strength, which was easily broken by the moisture, resulting in a low residual rate. In addition, the low-molecular-weight polysaccharose in the SF was soluble in water and further reduced the residual rate. When the HMP additive reached 9%, the residual rate was improved by 5.1% relative to that of the SF adhesive, reaching 83.5%. With the HMP additive further increased to 18%, the residual rate also increased by 5.4%, reached 88.9%; this increase was relative to that of the SF/9% HMP adhesive. The increased in residual rate could be attributed to the (i) reaction of HMP with polysaccharose and several soy proteins to form compact and homogeneous structures, which improved the cross-linking degree of the SF adhesive and reduced the number of hydrophilic groups, thereby blocking water intrusion; (ii) cross-linking of low-molecular-weight polysaccharose and formation of a network, which reduced the solubility of the polysaccharose; and (iii) formation of a dense interpenetrating network, which prevented water intrusion. All of these contributed to the increase in water resistance of the adhesive.

### 3.4. FTIR Spectroscopic Analysis

**Figure 7:**
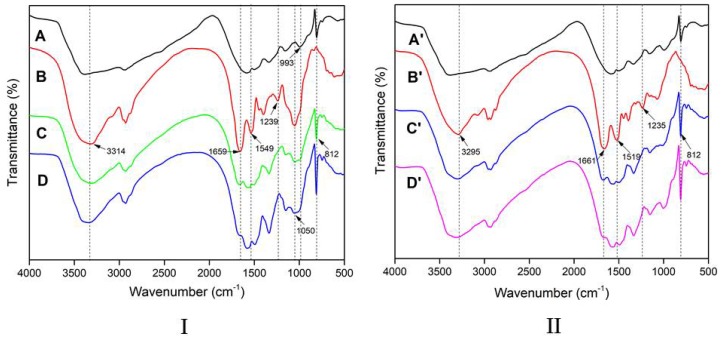
FTIR spectra of the samples: (**I**) (A (HMP), B (SF adhesive), C (SF/9% HMP adhesive), and D (SF/18% HMP adhesive)) and (**II**) (A’ (HMP), B’ (SPI adhesive), C’ (SPI/9% HMP adhesive), and D’ (SPI/18% HMP adhesive)).

The FTIR spectra of the HMP and different adhesives are presented in Figure 7. The peaks observed at approximately 3295–3314 cm^−1^ were assigned to the free bound O–H and N–H bending vibrations, which formed hydrogen bonds with the carbonyl group of the peptide linkage in soy protein [31]. The three major peaks of the soy protein were observed at 1659–1661, 1519–1549, and 1234–1239 cm^−1^, which were assigned to C=O stretching, N–H bending, C–N stretching, and N–H bending vibration, respectively [32,33]. In addition, the strongest absorption at 1050 cm^−1^ was observed in the SF adhesive, corresponding to C–O bending [34]. The peak at 993 cm^−1^ was attributed to the –OH out-of-plane bending vibration [35,36]. A strong and sharp peak was found at 812 cm^−1^, which was a characteristic of the triazine ring [37].

With 9% HMP additive in the adhesive formulation, the peak of C=O (Figure 7I) shifted from 1659 to 1667 cm^−1^ (blue shift), indicating that the adhesive formed a more stable chemical linkage and exhibited a more dense structure [38]. When HMP was cross-linked with SF and formed a dense network, the vibration of the functions required more energy, exhibiting a blue shift. The SF adhesives with 9% and 18% HMP, with absorption at 1050 and 993 cm^−1^, were weaker than the SF adhesive and HMP, respectively. This result was attributed to the reaction between the methylol group of HMP and active groups of the polysaccharose [39]. The appearance of a peak at 812 cm^−1^ in the SF/HMP and SPI/HMP adhesives indicated that HMP existed in the adhesive system. Furthermore, the stretching vibration peak at 812 cm^−^^1^ in the SF/18% HMP spectra was stronger than that of the SF/9% HMP, indicating that the HMP was well dispersed in the adhesive. With an increase in HMP additive, the peak at 1239 cm^−1^ (Figure 7I) was weaker than that of the SF adhesive (Figure 7II also shows a similar condition), which was attributed to the reaction of the methylol group with the –NH_2_ of soy protein. HMP was successfully introduced into the SF adhesive system because of the quick reaction of methylol melamine with polysaccharose and then with soy protein. This process formed a tightly crosslinked homogeneous system, and this was consistent with the changes in wet strength (Figure 5).

### 3.5. Thermogravimetric (TG) Analysis

**Figure 8:**
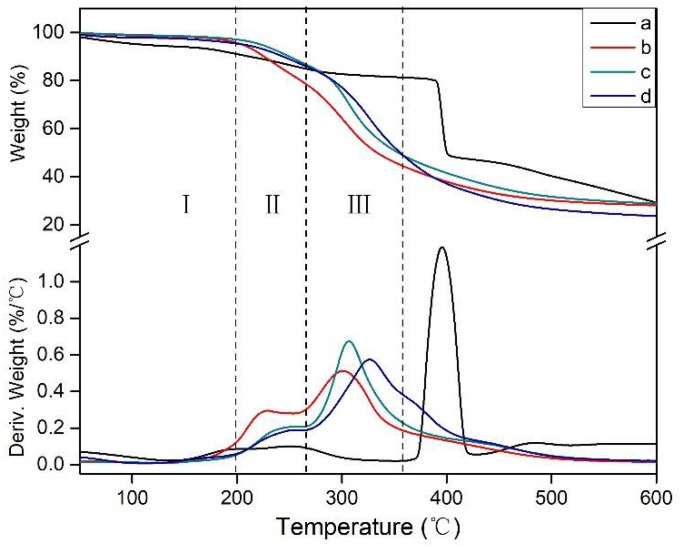
TG and DTG curves of adhesives: a (HMP), b (SF adhesive), c (SF/9% HMP adhesive), and d (SF/18% HMP adhesive). I: First stage, II: Second stage, III: Third stage.

In this study, TGA was used to evaluate the thermal stability of the different adhesives. The thermogravimetric (TG) and derivative thermogravimetric (DTG) curves of the adhesives are shown in Figure 8. The thermal degradation of the soy-based adhesive could be divided into three main stages (Table 3) [28,39]. After the third stage, further heating causes breakage of the C–C, C–N, and C–O linkages, and the backbone peptide bonds of soy protein decomposed, producing gases such as CO, CO_2_, NH_3_, and H_2_S [40].

A clear peak in the DTG curve of HMP was observed at 395.7 °C, indicating that HMP had a high degradation temperature. From the DTG curve associated with SF, two peaks at the second and third stages were attributed to the degradation of polysaccharose and soy protein, respectively [38]. When HMP was added into the SF adhesive, the peaks of the DTG curve at the second stage decreased, indicating the cross-linking of polysaccharose and formation of a denser network, which exhibited high thermal stability. Compared with the SF adhesive, the SF/HMP adhesive showed a weak peak at stage II, which was attributed to the formation of stable bonds by HMP and SF during curing of the resultant adhesive [41]. With an HMP additive in the adhesive formulation, the crosslinked polysaccharose network could play a major role in the SF/HMP adhesive system, which improved the water resistance of the adhesive. This finding was consistent with the adhesive residual rate analysis. In the third stage, with an HMP additive at 9%, the peak of the DTG curve increased to 306.8 °C compared with that of the SF adhesive (300.9 °C). This peak further moved to 326.5 °C when the HMP additive increased to 18%. In addition, no obvious peak was observed at 395.7 °C, suggesting a reaction between the HMP and SF. This reaction formed a denser cross-linked network than SF adhesive, which improved the thermal stability and water resistance of the adhesive.

### 3.6. SEM Analysis of Adhesives

**Figure 9:**
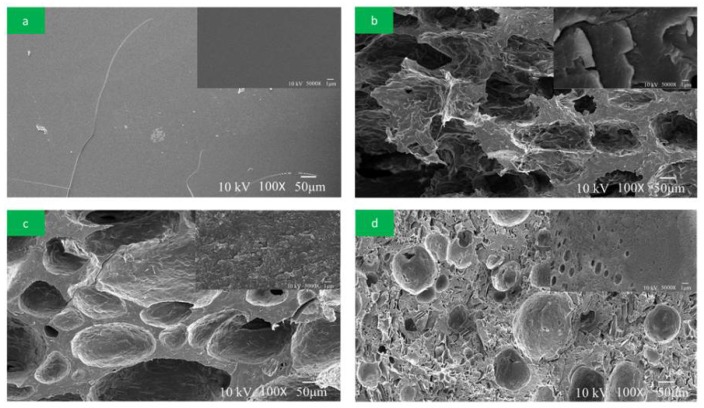
SEM images of the fracture surface: (**a**) (HMP), (**b**) (SF adhesive), (**c**) (SF/9% HMP adhesive), and (**d**) (SF/18% HMP adhesive).

The morphology of the fracture surfaces of the adhesives was examined by SEM to analyze the microscale surface deformation. Figure 9 presents the fracture surface of HMP and different adhesives. HMP obtained a highly uniform and dense structure after curing at 120 °C, which could effectively prevent the entry of moisture and result in pure HMP, exhibited excellent water resistance. Simultaneously, the SF adhesive clearly showed a loose and disordered fracture surface with numerous holes and cracks, which was mainly attributed to the uncrosslinked SF and the existence of water-soluble polysaccharose [19]. When the SF adhesive was cured at 120 °C, a large amount of water evaporated to form holes and cracks, which represented the channel of water intrusion. It caused the low water resistance and bad bond strength of the SF adhesive [28]. This result was in accordance with the study by Luo [13]. With an increase in HMP, the holes and cracks of the SF adhesive clearly decreased. With the HMP additive increased to 18%, the fracture surfaces of the SF adhesives appeared smoother and more compact. The reason was that HMP reacted with the SF and increased the density of the network structure during curing.

## 4. Conclusions

The HMP preferentially reacted with polysaccharose in SF than in soy protein. The wet shear strength of the plywood bonded with SF and SPI adhesives were 0.13 and 0.79 MPa, respectively. With 9% HMP additive, the shear strength of the plywood that bonded with the SF/HMP adhesive was 1.21 MPa, which was higher than the wet strength of the SPI/9%HMP adhesive (1.17 MPa). With a further increase in HMP additive to 12%, the wet shear strength of the SF/HMP adhesive was more significantly improved than that of the SPI/HMP adhesive.

The HMP additive in the adhesive improved the water resistance and bond performance of SF adhesives. With an increase in HMP additive, the residual rate continued to increase. When the HMP additive reached 9% and 15%, the residual rates were improved by 5.1% and 8.5%, compared with those of the SF. These increases led to final residual rates of 83.5% and 86.9%, respectively. With HMP additives of 9% and 15%, the wet shear strengths of the plywood were 1.21 MPa (63 °C) and 0.79 MPa (100 °C), which met the plywood requirements for interior and exterior uses, respectively. In addition, the shear strengths of the plywood bonded with the SF/PAE adhesive were 0.83 and 1.67 MPa immersed in water at 63 °C and placed in a dry room, respectively, which showed a poor performance that that of the plywood bonded with the SF/9% HMP adhesive (1.21 and 2.3 MPa). This improvement was attributed to the following: (i) The reaction of HMP with polysaccharose in SF and formation of cross-linked network; (ii) self-polycondensation of HMP; and (iii) formation of an interpenetrating network. This cross-linked network structure improved the thermal stability and formed a dense and smooth fracture surface, which contributed to the water resistance of the SF adhesive.

## Figures and Tables

**Table 1:** Various soy flour (SF) adhesive formulations.

Sample		SF Adhesive Formulations				
		SF (g)	WF (g)	Water (g)	HMP (g)	PAE (g)
**H**	HMP adhesive	-	20	40	40	-
**0**	SF adhesive	25	-	75	-	-
**1**	SF/3% HMP adhesive	25	-	69	3	-
**2**	SF/6% HMP adhesive	25	-	66	6	-
**3**	SF/9% HMP adhesive	25	-	63	9	-
**4**	SF/12% HMP adhesive	25	-	60	12	-
**5**	SF/15% HMP adhesive	25	-	57	15	-
**6**	SF/18% HMP adhesive	25	-	54	18	-
**7**	SF/21% HMP adhesive	25	-	51	21	-
**8**	SF/24% HMP adhesive	25	-	48	24	-
**8**	SF/24% HMP adhesive	25	-	48	24	-
**P**	SF/PAE adhesive	25	-	25	-	50 (12.5%)

**Table 2:** Various soy protein isolate (SPI) adhesive formulations.

Sample		SPI Adhesive Formulations		
		SPI (g)	Water (g)	HMP (g)
**0**	SPI adhesive	13.3	86.7	-
**a**	SPI/3% HMP adhesive	13.3	83.7	3
**b**	SPI/6% HMP adhesive	13.3	80.7	6
**c**	SPI/9% HMP adhesive	13.3	77.7	9
**d**	SPI/12% HMP adhesive	13.3	74.7	12

**Table 3:** Three main stages in thermal degradation of the SF adhesive.

Main Stages	Temperature Range	Changes in Thermal Degradation of the SF Adhesive
First stage	50–200 °C	A post-reaction stage, which is attributed to the possible reaction of the system under thermal action and produced vapor and gases, leading to a mass loss in the adhesive. The adhesive showed no degradation of the soy protein and other major components.
Second stage	200–270 °C	An initial degradation stage, which is mainly considered to the weight loss of the degradation of small molecules and the break of some unstable chemical bonds.
Third stage	270–360 °C	The degradation of the skeleton structure of the adhesive, which results from the thermal degradation of the cross-linked network structure in the adhesives.

## References

[1] Liu C., Zhang Y., Li X., Luo J., Gao Q., Li J. (2017). “Green” bio-thermoset resins derived from soy protein isolate and condensed tannins. Ind. Crops Prod..

[2] Wang F., Wang J., Chu F., Wang C., Jin C., Wang S., Pang J. (2018). Combinations of soy protein and polyacrylate emulsions as wood adhesives. Int. J. Adhes. Adhes..

[3] Mousavi S.Y., Huang J., Li K. (2018). Investigation of poly(glycidyl methacrylate-*co*-styrene) as a curing agent for soy-based wood adhesives. Int. J. Adhes. Adhes..

[4] Luo J., Luo J., Zhang J., Bai Y., Gao Q., Li J., Li L. (2016). A New Flexible Soy-Based Adhesive Enhanced with Neopentyl Glycol Diglycidyl Ether: Properties and Application. Polymers.

[5] Ghahri S., Mohebby B., Pizzi A., Mirshokraie A., Mansouri H.R. (2017). Improving Water Resistance of Soy-Based Adhesive by Vegetable Tannin. J. Polym. Environ..

[6] Liu C., Zhang Y., Li X., Luo J., Gao Q., Li J. (2017). A high-performance bio-adhesive derived from soy protein isolate and condensed tannins. RSC Adv..

[7] Li X., Li J., Luo J., Li K., Gao Q., Li J. (2017). A Novel Eco-friendly Blood Meal-based Bio-adhesive: Preparation and Performance. J. Polym. Environ..

[8] Fan D.-B., Qin T.-F., Chu F.-X. (2012). A Soy Flour-Based Adhesive Reinforced by Low Addition of MUF Resin. J. Adhes. Sci. Technol..

[9] He Z. (2017). Bio-Based Wood Adhesives: Preparation, Characterization, and Testing.

[10] Wang Z., Kang H., Zhang W., Zhang S., Li J. (2017). Improvement of Interfacial Adhesion by Bio-Inspired Catechol-Functionalized Soy Protein with Versatile Reactivity: Preparation of Fully Utilizable Soy-Based Film. Polymers.

[11] Zhao H.-J., Wang Y., Yang L.-L., Yuan L.-W., Peng D.-C. (2015). Relationship between phytoplankton and environmental factors in landscape water supplemented with reclaimed water. Ecol. Indic..

[12] Wang C., Wu J., Bernard G.M. (2014). Preparation and characterization of canola protein isolate–poly(glycidyl methacrylate) conjugates: A bio-based adhesive. Ind. Crops. Prod..

[13] Luo J., Li C., Li X., Luo J., Gao Q., Li J. (2015). A new soybean meal-based bioadhesive enhanced with 5,5-dimethyl hydantoin polyepoxide for the improved water resistance of plywood. RSC Adv..

[14] Cheng H.N., Ford C., Dowd M.K., He Z. (2017). Wood adhesive properties of cottonseed protein with denaturant additives. J. Adhes. Sci. Technol..

[15] Li X., Chen M., Zhang J., Gao Q., Zhang S., Li J. (2017). Physico-Chemical Properties of Soybean Meal-Based Adhesives Reinforced by Ethylene Glycol Diglycidyl Ether and Modified Nanocrystalline Cellulose. Polymers.

[16] Jang Y., Li K. (2015). An All-Natural Adhesive for Bonding Wood. J. Am. Oil Chem. Soc..

[17] Lawlor C.M., Riley C.A., Hildrew D.M., Guarisco J.L. (2015). Respiratory failure after superior-based pharyngeal flap for velopharyngeal insufficiency: A rare complication. Int. J. Pediatr. Otorhinolaryngol..

[18] Zhang X., Zhu Y., Yu Y., Song J. (2017). Improve Performance of Soy Flour-Based Adhesive with a Lignin-Based Resin. Polymers.

[19] Luo J., Li L., Luo J., Li X., Li K., Gao Q. (2016). A High Solid Content Bioadhesive Derived from Soybean Meal and Egg White: Preparation and Properties. J. Polym. Environ..

[20] Yuan C., Luo J., Luo J., Gao Q., Li J. (2016). A soybean meal-based wood adhesive improved by a diethylene glycol diglycidyl ether: Properties and performance. RSC Adv..

[21] Li H., Li C., Gao Q., Zhang S., Li J. (2014). Properties of soybean-flour-based adhesives enhanced by attapulgite and glycerol polyglycidyl ether. Ind. Crops Prod..

[22] Guo M., Li W., Han N., Wang J., Su J., Li J., Zhang X. (2018). Novel Dual-Component Microencapsulated Hydrophobic Amine and Microencapsulated Isocyanate Used for Self-Healing Anti-Corrosion Coating. Polymers.

[23] Banu H.T., Meenakshi S. (2017). Synthesis of a novel quaternized form of melamine–formaldehyde resin for the removal of nitrate from water. J. Water Process Eng..

[24] Zhu P., Gu Z., Hong S., Lian H. (2018). Preparation and characterization of microencapsulated LDHs with melamine-formaldehyde resin and its flame retardant application in epoxy resin. Polym. Adv. Technol..

[25] Wang Y., Xie Y., Zhang Y., Tang S., Guo C., Wu J., Lau R. (2016). Anionic and cationic dyes adsorption on porous poly-melamine-formaldehyde polymer. Chem. Eng. Res. Des..

[26] Yin D., Ma L., Geng W., Zhang B., Zhang Q. (2015). Microencapsulation of n-hexadecanol by in situpolymerization of melamine-formaldehyde resin in emulsion stabilized by styrene-maleic anhydride copolymer. Int. J. Energy Res..

[27] Chen N., Zheng P., Zeng Q., Lin Q. (2017). Characterization and Performance of Soy-Based Adhesives Cured with Epoxy Resin. Polymers.

[28] Li J., Luo J., Li X., Yi Z., Gao Q., Li J. (2015). Soybean meal-based wood adhesive enhanced by ethylene glycol diglycidyl ether and diethylenetriamine. Ind. Crops Prod..

[29] Zheng P., Lin Q., Li F., Ou Y., Chen N. (2017). Development and characterization of a defatted soy flour-based bio-adhesive crosslinked by 1,2,3,4-butanetetracarboxylic acid. Int. J. Adhes. Adhes..

[30] Luo J., Luo J., Bai Y., Gao Q., Li J. (2016). A high performance soy protein-based bio-adhesive enhanced with a melamine/epichlorohydrin prepolymer and its application on plywood. RSC Adv..

[31] Luo J., Li X., Zhang H., Gao Q., Li J. (2016). Properties of a soybean meal-based plywood adhesive modified by a commercial epoxy resin. Int. J. Adhes. Adhes..

[32] Luo J., Luo J., Li X., Li K., Gao Q., Li J. (2016). Toughening improvement to a soybean meal-based bioadhesive using an interpenetrating acrylic emulsion network. J. Mater. Sci..

[33] Li X., Luo J., Gao Q., Li J. (2016). A sepiolite-based united cross-linked network in a soybean meal-based wood adhesive and its performance. RSC Adv..

[34] Zhang L., Hu Y., Duan X., Tang T., Shen Y., Hu B., Liu A., Chen H., Li C., Liu Y. (2018). Characterization and antioxidant activities of polysaccharides from thirteen boletus mushrooms. Int. J. Biol. Macromol..

[35] Song Y., Ma R., Jiao C., Hao L., Wang C., Wu Q., Wang Z. (2017). Magnetic mesoporous polymelamine-formaldehyde resin as an adsorbent for endocrine disrupting chemicals. Mikrochim. Acta.

[36] Li J., Li Q., Li L.-S., Xu L. (2017). Removal of perfluorooctanoic acid from water with economical mesoporous melamine-formaldehyde resin microsphere. Chem. Eng. J..

[37] Wu X., Shi Z., Tjandra R., Cousins A.J., Sy S., Yu A., Berry R.M., Tam K.C. (2015). Nitrogen-enriched porous carbon nanorods templated by cellulose nanocrystals as high performance supercapacitor electrodes. J. Mater. Chem. A.

[38] Yuan C., Chen M., Luo J., Li X., Gao Q., Li J. (2017). A novel water-based process produces eco-friendly bio-adhesive made from green cross-linked soybean soluble polysaccharide and soy protein. Carbohydr. Polym..

[39] Yang Y., Cui S.W., Gong J., Guo Q., Wang Q., Hua Y. (2015). A soy protein-polysaccharides Maillard reaction product enhanced the physical stability of oil-in-water emulsions containing citral. Food Hydrocoll..

[40] Li J., Li X., Li J., Gao Q. (2015). Investigating the use of peanut meal: A potential new resource for wood adhesives. RSC Adv..

[41] Luo J., Luo J., Yuan C., Zhang W., Li J., Gao Q., Chen H. (2015). An eco-friendly wood adhesive from soy protein and lignin: Performance properties. RSC Adv..

